# miR-195 inhibits macrophages pro-inflammatory profile and impacts the crosstalk with smooth muscle cells

**DOI:** 10.1371/journal.pone.0188530

**Published:** 2017-11-22

**Authors:** Joao Paulo Bras, Andreia Machado Silva, George A. Calin, Mario Adolfo Barbosa, Susana Gomes Santos, Maria Ines Almeida

**Affiliations:** 1 Instituto de Investigação e Inovação em Saúde, Universidade do Porto, Porto, Portugal; 2 Instituto de Engenharia Biomédica, Universidade do Porto, Porto, Portugal; 3 Faculdade de Engenharia da Universidade do Porto, Universidade do Porto, Porto, Portugal; 4 Instituto de Ciências Biomédicas Abel Salazar, Universidade do Porto, Porto, Portugal; 5 The University of Texas MD Anderson Cancer Center, Houston, Texas, United States of America; Qatar University College of Health Sciences, QATAR

## Abstract

Macrophages are a main component of atherosclerotic plaques. Recent studies suggest that pro-inflammatory M1 macrophages are pro-atherogenic while M2 macrophages promote plaque stability. Moreover, toll-like receptor signalling pathways are implicated in atherosclerotic plaque formation, evolution and regression. We propose microRNAs as key regulators of these processes. In this context, our goal is to promote inflammation resolution using miR-195 to reduce M1-like macrophage polarization and to evaluate the molecular mechanisms underlying such effect, as well as to explore the functional consequences for smooth muscle cell recruitment. Human primary macrophages were differentiated from peripheral blood monocytes and stimulated with LPS or IL-10 to promote M1 or M2c polarization, respectively. miR-195 levels were upregulated in M2c macrophages compared with M1 macrophages. In THP-1 macrophages stimulated with LPS and IFN-γ, results show that TLR2 levels were reduced by miR-195 overexpression compared with scrambled control. In addition, phosphorylated forms of p54 JNK, p46 JNK and p38 MAPK were decreased by miR-195 in macrophages following M1 stimulation. Moreover, miR-195 significantly decreased levels of IL-1β, IL-6 and TNF-α pro-inflammatory cytokines in the supernatants of M1-stimulated macrophage cultures. At the functional level, results from smooth muscle cell recruitment and migration models showed that miR-195 impairs the capacity of M1 macrophages to promote smooth muscle cells migration. In conclusion, miR-195 is involved in macrophage polarization and inhibits TLR2 inflammatory pathway mediators. Moreover, miR-195 impairs the effect of macrophages on smooth muscle cells recruitment capacity and migration profile. Thus, miR-195 might be used as a new potential tool to promote inflammation resolution in cardiovascular research.

## Introduction

Chronic inflammation is a major feature of atherosclerosis, which underlies several cardiovascular diseases [1,2]. In atherosclerosis, the normal homeostatic functions of the endothelium are altered, promoting an inflammatory response. Initially, adhesion molecules expressed by inflamed endothelium recruit leukocytes, mainly monocytes, which penetrate into the intima and differentiate into macrophages, predisposing the vessel wall to lipid accretion or vasculitis [3]. Many of the inflammatory processes that occur throughout atherogenesis are orchestrated by resident and recruited macrophages [4,5]. These cells can adopt diverse activation states in response to micro-environmental triggers [6].

Macrophage heterogeneity is particularly important in atherosclerosis lesions due to the opposing roles of M1 (pro-inflammatory) and M2 (anti-inflammatory) macrophage phenotypes, which are dependent on the inflammatory molecules present in the microenvironment. During disease progression, the increase in the total number of macrophages and the presence of pro-inflammatory cytokines has an enormous impact in increased plaque vulnerability [7]. In the lesion site most prone to plaque rupture, macrophages expressing pro-inflammatory markers are shown to be the most predominant population, while in the fibrous cap regions, M2 macrophages are shown to have greater impact [8]. Thus, although potential deleterious effects of M1 macrophages could be counteracted by the protective pro-fibrotic and tissue repair effects of M2 macrophages in the fibrous cap, the limited number of M2 macrophages in the plaque cannot balance the M1-mediated effects, promoting plaque instability [8,9]. The emerging understanding about macrophage interactions with vascular smooth muscle cells (VSMC) in the atherosclerotic plaque, and how macrophage secretion profile influence VSMC phenotype, led to the general consensus that pro-inflammatory M1 macrophages are pro-atherogenic, while M2 macrophages may promote plaque stability [6,10,11]. Therefore, modulation of macrophage function by reducing their pro-inflammatory activity in order to promote plaque stability is an exciting therapeutic prospect.

microRNAs (miRNAs) are a class of small non-coding RNAs (approximately 15–25 nucleotides long) crucial for post-transcriptional regulation of gene expression. Mature miRNAs bind to 3′untranslated region (UTR), coding regions or 5′UTR of messenger RNAs (mRNAs), inhibiting translation or causing mRNA degradation [12]. Importantly, miRNAs are involved in key cellular functions, including macrophage polarization. Thus, miRNAs are able to modulate the magnitude of an inflammatory response by targeting mRNA encoding cytokines, surface receptors and other inflammation-related molecules, which have direct consequences in macrophage phenotypes. Recently, several miRNAs have been shown to be involved in pathways that induce M1 (miR-9, miR-127 and miR-155) [13–15] and M2 (miR-125a-5p, miR-146a, miR-223 and let-7c) [16–18] macrophage polarizations, while others are directly involved in the promotion and aggravation of atherosclerosis. Interestingly, miR-195 inhibits VSMC proliferation and migration, and reduces neointimal formation in a balloon-injured carotid artery [19], which shows that miR-195 plays a role in the cardiovascular system.

A single miRNA is able to regulate a large number of mRNAs [12] and potentially regulate simultaneously inflammation and atherosclerosis processes, which encourages their use as therapeutic targets in the context of atherosclerosis lesions. The aim of this study is to evaluate the role of miR-195 in inflammation resolution through modulation of macrophage polarization and to determine the consequent functional effect on their crosstalk with other cellular components of the plaque, namely smooth muscle cells.

## Materials and methods

### Ethics statement

In this study, human primary monocytes were isolated from buffy coats of healthy blood donors, obtained through a collaboration protocol with *Serviço de Imunohemoterapia*, *Centro Hospitalar São João (CHSJ)*. This was approved by CHSJ Ethics Committee for Health (References 259 and 260/11), and conforms to the declaration of Helsinki. A written informed consent was obtained from all subjects before blood donation. Buffy coats were provided anonymized, and their identification was not accessible to researchers.

### Human primary monocyte isolation, differentiation and polarization

Human primary monocytes were obtained from healthy blood donors buffy coats (BC). RosetteSep human monocyte enrichment isolation kit (StemCell Technologies) was used, according to Oliveira *et al*. [20]. Firstly, BC were centrifuged at room temperature (RT) for 20 minutes (min) at 1200 g, without active acceleration or brake, for blood components separation. Peripheral blood mononuclear cells (PBMCs) layer, and red blood cells necessary to the formation of immunorosettes, were collected and incubated with RosetteSep human monocyte enrichment isolation kit (Stemcell Technologies) for 20 min, under gentle mixing. The mixture was diluted at a 1:1 ratio with 2% fetal bovine serum (FBS) in phosphate buffered saline (PBS), gently layered over Histopaque-1077 (Sigma) and centrifuged as described above. The enriched monocyte layer was collected, and washed with PBS for platelet depletion, by centrifugation at 700 rpm for 17 min. Recovered monocytes were plated (0.5 x 10^6^ cells/well) on top of sterile 13 mm glass coverslips in 24-well plates and cultured in complete culture medium (RPMI 1640, Life Technologies) supplemented with 10% FBS (Lonza) and 1% penicillin streptomycin (P/S, Invitrogen), in a humidified 37°C/5% CO_2_ incubator. Primary human monocytes were allowed to differentiate into macrophages. Cell culture media was carefully changed at day 7, ensuring minimum cell disturbance and incubated for 3 additional days. At day 10, polarization of differentiated macrophages was induced. Cells were treated with 10 ng/mL lipopolysaccharide (LPS) to stimulate pro-inflammatory M1 macrophages, or with 10 ng/mL interleukine-10 (IL-10) to induce anti-inflammatory M2c macrophages, and incubated for 3 additional days.

### THP-1 monocyte differentiation and polarization

THP-1 monocytic cell line [21] was cultured in complete RPMI 1640 medium supplemented with 10% FBS and 1% P/S. In order to induce a macrophage-like phenotype, cells were plated in 24-well plates (0.25 x 10^6^ cells/well) with medium containing 100 ng/mL of phorbol 12-myristate 13-acetate (PMA) (Sigma) and allowed to differentiate for 48h. To obtain macrophages at a resting state, adherent cells were cultured in medium without PMA for additional 24 h. To mimic a pro-inflammatory environment and consequently promote an M1 polarization, cells were stimulated with 100 ng/mL LPS and 20 ng/mL IFN-γ (Sigma).

### Human primary umbilical artery smooth muscle cells

Human primary umbilical artery smooth muscle cells (HUASMC), kindly donated by Dr. Elisa Cairrão from *Centro de Investigação em Ciências da Saúde*, *Universidade da Beira Interior* (Portugal), were cultured in Dulbecco’s modified Eagle’s F12 (DMEM-F12) media supplemented with 5% FBS, epidermal growth factor (EGF, 5 μg/mL), fibroblast growth factor (FGF, 0.5 ng/mL), heparin (2 μg/mL) (all from Immunotools), and insulin (5 μg/mL) (Sigma). Cells were plated in T75 flasks previously coated with collagen type I (Sigma) and cultured in a humidified 37°C/5% CO_2_ incubator.

### Transfections

Differentiated THP-1 macrophages were transfected with miRNA mimics (Pre-miR miRNA Precursor miR-195-5p) or Pre-miR miRNA Precursor Negative Control (Scrambled—SCR) (Life Technologies), using Lipofectamine 2000 transfection reagent (Invitrogen), as recommended by the manufacturer. Final miRNA mimics concentration was 50 nM per well. Non-transfected cells were analyzed simultaneously as a control.

### RNA extraction

Total RNA was extracted from macrophages using TRIzol reagent (Invitrogen) according to the manufacturer’s instructions. RNA concentration and purity was evaluated by measuring absorbance in NanoDrop 1000. Ratio of 260/280 nm and 260/230 nm range was between 1.8 and 2. RNA integrity was accessed by gel electrophoresis.

### Reverse transcription and real-time quantitative polymerase chain reaction

miRNA expression was evaluated in primary and THP-1 macrophages by Reverse Transcription—Real-Time quantitative Polymerase Chain Reaction (RT-qPCR) using TaqMan miRNA assays (Applied Biosystems). Firstly, cDNA was synthesized using RNA, TaqMan MicroRNA Reverse Transcription Kit (Applied Biosystems) and gene specific stem-loop Reverse Transcription primers (hsa-miR-195-5p and small nuclear RNA U6) (Applied Biosystems). qPCR reactions were prepared using cDNA, miR-195 or small nuclear RNA U6 TaqMan probe (Applied Biosystems) and SsoAdvanced^™^ Universal Probes Supermix (Bio-Rad).

For gene expression array data validation, RNA from SCR and miR-195 transfected THP-1 macrophages was treated with TURBO DNA-free Kit (Invitrogen) and complementary DNA (cDNA) was synthesized using Random Hexamers (Invitrogen), dNTPs (Bioline) and SuperScript^®^ III Reverse Transcriptase (Invitrogen). qPCR was carried out using cDNA, primers and iQ SYBR Green Supermix (Bio-Rad). Primers used for qPCR experiments are shown in S1 Table.

qPCR reactions were performed in duplicate in a MyCycler Thermal Cycler (Bio-Rad) and relative expression levels were calculated using the quantification cycle (Cq) method, according to MIQE guidelines [22].

### Flow cytometry

Differentiated and polarized macrophages derived from primary monocytes or THP-1 cell line were harvested with 5mM EDTA-PBS and centrifuged at 300 g for 5 min, at 4°C. Cells were resuspended in FACS buffer (PBS 1X, 2% FBS, 0.01% sodium azide) and immunostained for cell markers with fluorochrome-conjugated antibodies, for 20 min at 4°C in the dark. For primary human macrophages, CD14 was used as lineage marker [20] and CD86 and CD163 were used as M1 and M2 specific markers, respectively. The following antibodies were used: anti-CD14-APC (Immunotools), anti-CD86-FITC (Immunotools) and anti-CD163-PE (BD Biosciences Pharmingen). Unlabeled macrophages and labelling with isotypes IgG1-APC, IgG1-FITC and IgG1-PE (all from Immunotools) were used as negative controls.

To evaluate levels of toll-like receptors (TLRs) in THP-1 transfected macrophages the following antibodies were used: anti-CD11b-PE (eBioscience), anti-TLR4-Alexa Fluor 488 (eBioscience) and anti-TLR2-Alexa Fluor 647 (Biolegend). Unlabeled THP-1 macrophages and isotypes immunostaining with IgG1-PE, IgG2-FITC and IgG1-APC (all from Immunotools) were used as control. To evaluate intracellular TLR expression levels, cells were additionally permeabilized for 5 min with 0.1% Triton in PBS, blocked with 1% Bovine Serum Albumin (BSA) in PBS and then immunostained using the cocktail of antibodies described above (containing anti-CD11b-PE, anti-TLR4-Alexa Fluor 488 and anti-TLR2-Alexa Fluor 647). Non-transfected, non-stimulated THP-1 macrophages were used as control.

Fluorescence was measured in FACS Canto II flow cytometer (BD Biosciences Pharmingen) with BD FACS Diva software. Results were analyzed using FlowJo Software V10 (FlowJo, LLC).

### Western blot

THP-1-derived macrophages were transfected with miR-195 mimics or SCR and then stimulated with 100 ng/mL LPS and 20 ng/mL IFN-γ. Cells were then harvested and washed twice with cold PBS before cell lysis with RIPA buffer in the presence of protease and phosphatase inhibitors. Lysates were centrifuged (14000 rpm, 10 min, 4°C) and protein was quantified using DC protein assay kit (Bio-Rad). Protein samples were resolved by SDS-PAGE in reducing conditions and transferred to nitrocellulose membranes, which were blocked in a solution of 5% BSA in TBS-Tween 0.1% or 5% milk in TBS-Tween 0.1% for phosphorylated or total proteins detection, respectively. Membranes were then probed overnight using the following primary antibodies: anti-phospho-p38 MAP Kinase (Thr180/Tyr182), anti-phospho-SAPK/JNK (Thr183/Tyr185), anti-p38 MAP Kinase, anti-GAPDH, anti-α-tubulin (all from Cell Signaling) and anti- JNK (D-2) (Santa Cruz Biotechnology); appropriate secondary antibodies conjugated to horseradish peroxidase (HRP) were used for signal detection, followed by membrane incubation with chemiluminescent substrate (ECL, GE Healthcare) and exposure to X-ray films.

### Enzyme-linked immunosorbent assay (ELISA)

Supernatants of transfected/stimulated macrophage cultures were collected and levels of cytokines were evaluated using enzyme-linked immunosorbent assay (ELISA), according to the manufacturer’s protocol, namely: IL-1β (Legend Max Human ELISA kits, BioLegend), IL-6, and TNF-α (Human Standard ABTS ELISA Development Kits, PeproTech). Cytokine concentrations (pg/mL) were determined using a standard calibration curve.

### Migration assays

Evaluation of miR-195 effect on the capacity of THP-1 macrophages to recruit HUASMC was assessed by transwell assays. Firstly, THP-1 cells were differentiated to macrophages, transfected with either miR-195 or SCR, and stimulated with 100 ng/mL LPS and 20 ng/mL IFN-γ for six hours, in 24-well plates (bottom compartment of the transwell system—BC). Then, HUASMC in FBS-free media were plated in Boyden chambers (inserts) with PET membranes of 8 μm pore size, previously coated with sterile 1% BSA (top compartment of the transwell system–TC). Finally inserts were placed on top of the wells containing THP-1 derived cells, and HUASMC were allowed to migrate through the membrane for 24h. M1 stimulation cytokines only, non-stimulated, and non-transfected THP-1 macrophages were used as controls for HUASMC migration. Then, culture media was removed and membranes washed twice with PBS. Cells were fixed with 4% paraformaldehyde for 15 min at RT. Insert membranes were washed again with PBS and the inner side of the membrane was scrubbed with a cotton swab to remove non-migrated cells. Membrane was removed from the insert and mounted on a microscope slide using Fluoroshield with DAPI (Sigma). Six random fields of migrated nuclei were counted per membrane and fold-change in migration was calculated relative to non-stimulated, non-transfected THP-1 macrophages.

For wound healing assays, HUASMC were plated in 24 well plates pre-coated with collagen type I (0.15x10^5^ cells/m^2^). Cells were cultured in DMEM F12 containing 10% FBS until they reached 80% confluence. Then, the monolayer was disrupted in the middle of the well, using a plastic tip, to create a “wound”. Cells were then washed with PBS 1x and cultured with conditioned media from differently stimulated THP-1 macrophages, up to 36 hours. Images of the wound area were obtained at 0, 12, 24 and 36 hours using a 5x objective, and gap area quantified using Fiji Software to evaluate HUASMC migration.

### *In silico* miR-195 targets prediction

*In silico* predictions of miR-195-5p targets were performed by screening the databases TargetScan (http://www.targetscan.org/), RNA22 (https://cm.jefferson.edu/rna22/Interactive/), miRanda (http://www.microrna.org/microrna/home.do) and miRWalk (http://zmf.umm.uni-heidelberg.de/apps/zmf/mirwalk2/). hsa-miR-195-5p sequence annotation was obtained from the miRBase database (http://www.mirbase.org/). Then, from the list of mRNAs predicted to be targeted by miR-195, the interaction with TLR2 was specifically analyzed. Target genes transcripts FASTA format sequence annotations were obtained from Ensemble (http://www.ensembl.org/).

### Luciferase assay

Cloning and luciferase assays were performed as previously described [23]. Briefly, TLR2 mRNA fragments of about 200 nt that contained the miR-195 putative binding site were amplified by PCR using primers containing the XbaI restriction enzyme site. PCR products were purified, digested, and directly cloned into the Xbal site of the pGL3 control vector (Promega Corporation, Madison, WI), located downstream of the firefly luciferase reporter gene. The QuikChange II XL site-directed mutagenesis kit (Agilent Technologies, Santa Clara, CA) was used to generate mutations in the miRNA-binding site. All primers are listed in S2 Table. Cells were cotransfected with 50 nM of SCR or miR-195, and 0.4 μg of pGL3-putative binding site plasmids or pGL3-mutated putative binding site plasmids, together with Renilla luciferase construct that was used as a normalization reference. Transfections were performed in OPTI-MEM I (Invitrogen) using Lipofectamine 2000 reagent (Invitrogen). Cells were lysed 48 h after transfection and luciferase activity was measured in Veritas Microplate Luminometer (Turner Biosystems), using a dual-luciferase reporter assay system (Promega Corporation), according to the manufacturer protocol.

### cDNA microarray and gene functional annotation

RNA from miR-195-THP1 macrophages and SCR-THP-1 macrophages was isolated as described above and RNA quality was assessed using Bioanalyzer RNA 6000 Nano Kit (Agilent). Gene expression was determined using GeneChip Human Clariom S arrays (Affymetrix). Results were analysed using Transcriptome Analysis Console (TAC) Software (Affymetrix). Ingenuity Pathway Analysis (IPA; https://www.qiagenbioinformatics.com/products/ingenuitypathway-analysis) was performed to identify the pathways and biological functions associated to miR-195 downregulated genes.

### Statistical analysis

Statistical analysis was performed using GraphPad Prism version 7 (GaphPad Software, Inc.). For data that passed the normality test, paired t-test or one-way ANOVA were used to compare the samples. When data did not pass the normality test, non-parametric test of Friedman Test, followed by uncorrected Dunn’s multiple comparison test, comparing ouser defined sets of data, were used to evaluate significant differences between the different samples. Statistical significance was considered for p<0.05 (* p<0.05, ** p<0.01, *** p<0.001, **** p<0.0001, n.s.: non-significant).

## Results

### miR-195 is increased in human M2 macrophages

**Fig 1:**
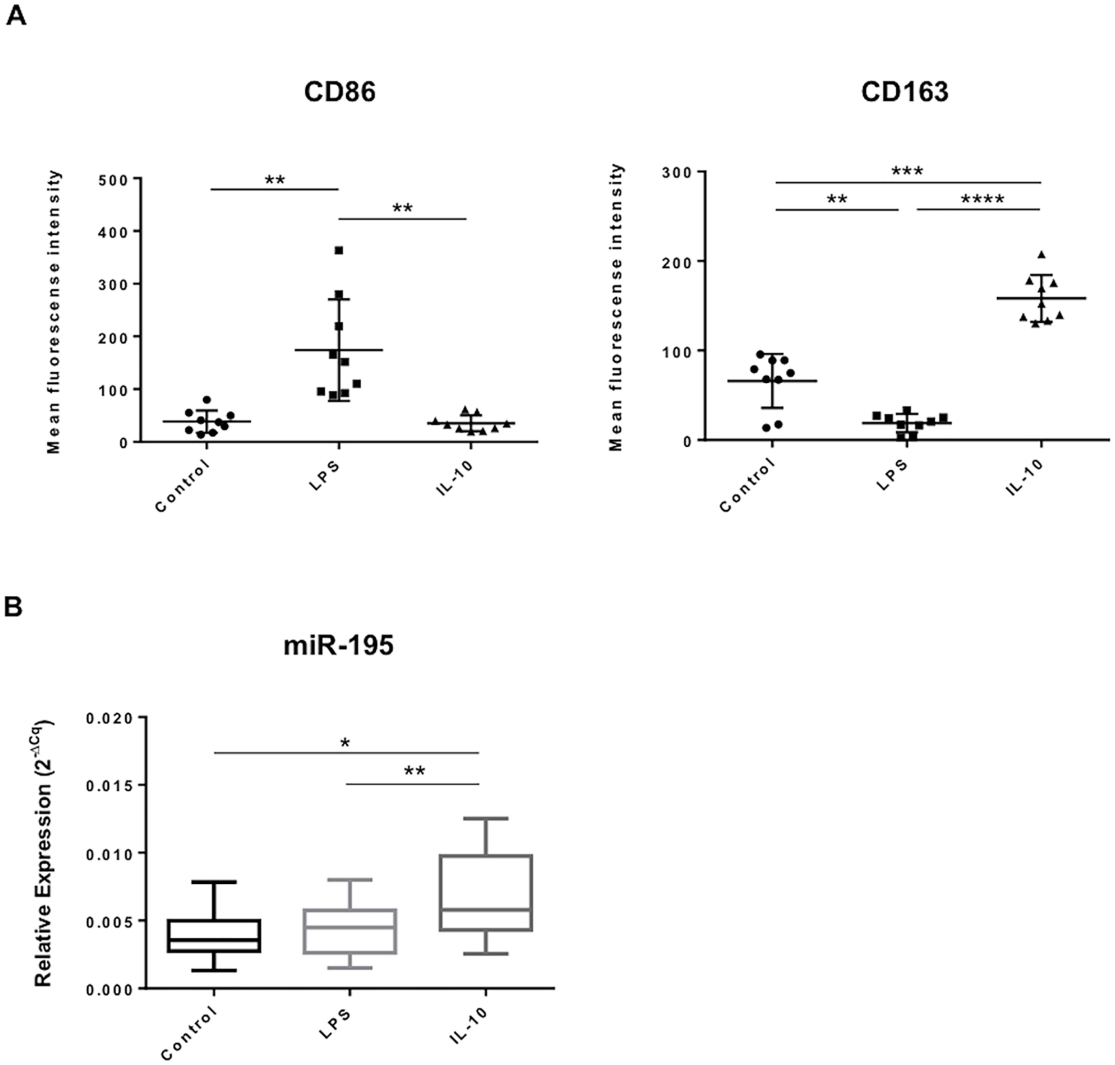
miR-195 is overexpressed in IL-10-treated macrophages. Human primary monocytes were differentiated into macrophages for 10 days and further polarized for 3 days in presence of LPS or IL-10. A) Surface expression levels of M1 marker CD86 (left) and the M2 marker CD163 (right) in LPS or IL-10 stimulated macrophages, respectively, assessed by flow cytometry (mean fluorescence intensity ± SD, n = 9). B) Relative miR-195 expression levels in non-treated, LPS and IL-10 treated macrophages, determined by RT-qPCR (n = 9); Cq: quantification cycle. Statistical significance: *p<0.05, **p<0.01, ***p<0.001, ****p<0.0001.

In order to investigate the role of miR-195 on macrophage polarization phenotypes relevant to atherosclerosis, levels of miR-195 were evaluated upon macrophage treatment with different stimuli to induce alterations in macrophage polarization status. Primary human monocytes were successfully isolated and differentiated into macrophages, as shown by optical microscopy observation and flow cytometry (S1A and S1B Fig). More than 90% of the cells were positive for CD14, the macrophage lineage cell marker, indicating high purity of the cell population after isolation (S1B Fig). Next, to induce macrophage polarization into pro-inflammatory M1 or anti-inflammatory M2c phenotypes, cells were treated with 10 ng/mL LPS or 10 ng/mL IL-10, respectively. Macrophage polarization was evaluated by flow cytometry for cell surface expression of M1 marker CD86 and M2c marker CD163. Results from 9 independent donors show, as expected, significantly higher mean fluorescence intensity (MFI) levels of cell surface marker CD86 in LPS-treated macrophages (MFI = 173.9 ± 96.1) compared with IL-10-treated macrophages (MFI = 35.2 ±15.1; p<0.01) or with the non-treated macrophages (MFI = 38.4 ± 21.0; p<0.01) (Fig 1A, left plot; S1C Fig). On the other hand, IL-10-treated cells show significantly higher levels of CD163 (MFI = 158.3 ± 26.2) compared with LPS-treated cells (MFI = 18.9±10.3; p<0.0001) and non-treated control group (MFI = 66.0 ± 30.2; p<0.001) (Fig 1A, right plot; S1C Fig). Moreover, levels of CD163 are significantly reduced in LPS-treated macrophages compared with control (p<0.01) (Fig 1A, right plot). Finally, miR-195 expression levels were evaluated by RT-qPCR in M1 and M2c macrophage phenotypes. Results show that miR-195 levels are significantly higher in IL-10-treated macrophages, when compared with LPS-treated and non-treated control macrophages (p<0.05) (Fig 1B). Therefore, miR-195 is involved in the response of macrophages to M2c stimuli.

### miR-195 reduces TLR2 levels in macrophages

**Fig 2:**
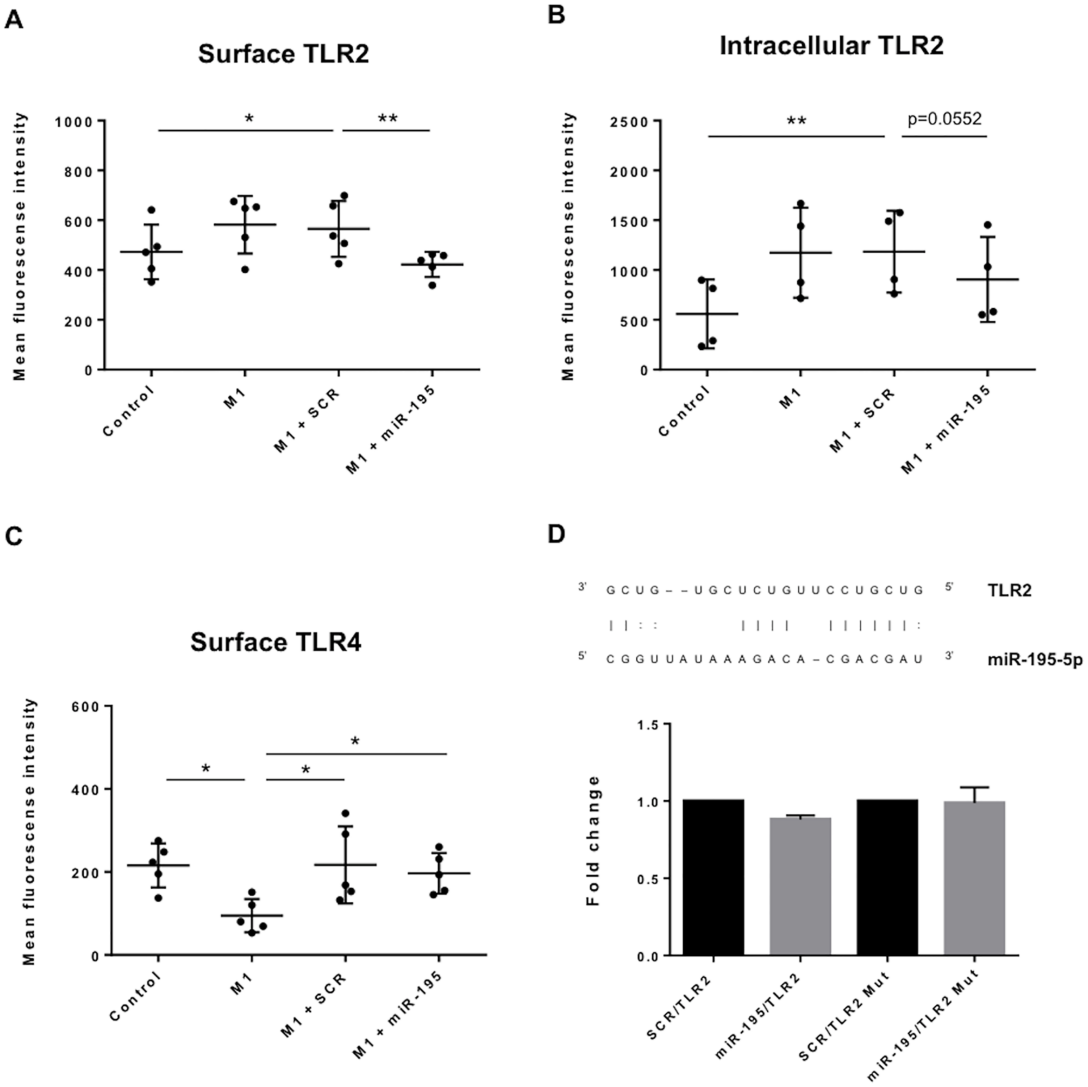
miR-195 impacts TLR2 surface and intracellular levels upon pro-inflammatory stimuli. A) Flow cytometry analysis of TLR2 surface (n = 5) and B) intracellular (n = 4) levels on THP-1 macrophages after transfection with either SCR or miR-195 and after induction of M1-like macrophages with LPS and IFN-γ. C) TLR4 surface levels on THP-1 macrophages were also evaluated by flow cytometry (n = 5). D) *In silico* target prediction for hsa-miR-195-5p interaction with TLR2 mRNA; graphic shows normalized levels of luciferase activity of a representative experiment performed in triplicates (mean ± SD); Control: Non-transfected, non-stimulated THP-1 macrophages; Mutated (Mut): vector with a deletion in the predicted binding site. Statistical significance: *p<0.05, **p<0.01.

TLR activation is implicated in atherogenesis, as it initiates signaling cascades that culminate in the production of pro-inflammatory cytokines, foam cell formation and activation of adaptive immunity [24–26]. Therefore, we explored the impact of miR-195 in the expression of TLRs known to be involved in human atherosclerotic plaques formation, namely TLR2 and TLR4. To address this, gain-of-function studies were performed in THP-1 macrophages. Transfections of miR-195 mimics were successfully performed, as shown by RT-qPCR results (p<0.0001) (S2 Fig). Results show that TLR2 levels are significantly increased at the cell surface (p<0.05) and intracellularly (p<0.01) of SCR-transfected cells, upon stimulation for M1 phenotype compared with control (Fig 2A and 2B). Importantly, under pro-inflammatory conditions, cells overexpressing miR-195 show a significant reduction in TLR2 at the cell surface and intracellularly compared with M1 macrophages transfected with SCR (p<0.01) (Fig 2A). Similarly, TLR2 intracellular levels are decreased by miR-195-overexpressing cells compared with SCR in a pro-inflammatory environment (p = 0.0552). Levels of TLR4 were also evaluated, and results show a significant decrease of cell surface TLR4 upon M1 stimulation (Fig 2C), indicating that this receptor is internalized after LPS binding (S3 Fig), in agreement with previous work by us and others [27–29]. However, macrophages overexpressing miR-195 do not significantly change their surface or intracellular TLR4 levels compared with SCR-transfected macrophages. (Fig 2C; S3 Fig). In conclusion, miR-195 specifically impacts TLR2 but not TLR4 levels in a pro-inflammatory environment.

Following these results, we hypothesized that miR-195 could influence TLR2 expression by directly targeting its mRNA. To address this hypothesis, we followed an unbiased bioinformatics approach whereby prediction databases for miRNA-mRNA interaction were screened and tested for complementary base pair interaction between miR-195 and TLR2 mRNA. TLR2 was predicted to be a target of miR-195 by RNA22 and miRWalk algorithms (Fig 2D). To confirm the interaction, luciferase assays were performed with and without mutation of the predicted binding site. However, the results obtained show no direct interaction between miR-195 and TLR2 for the predicted binding site (Fig 2D), suggesting TLR2 expression is regulated by miR-195 through an indirect mechanism.

### miR-195 inhibits the TLR2 signaling pathway

**Fig 3:**
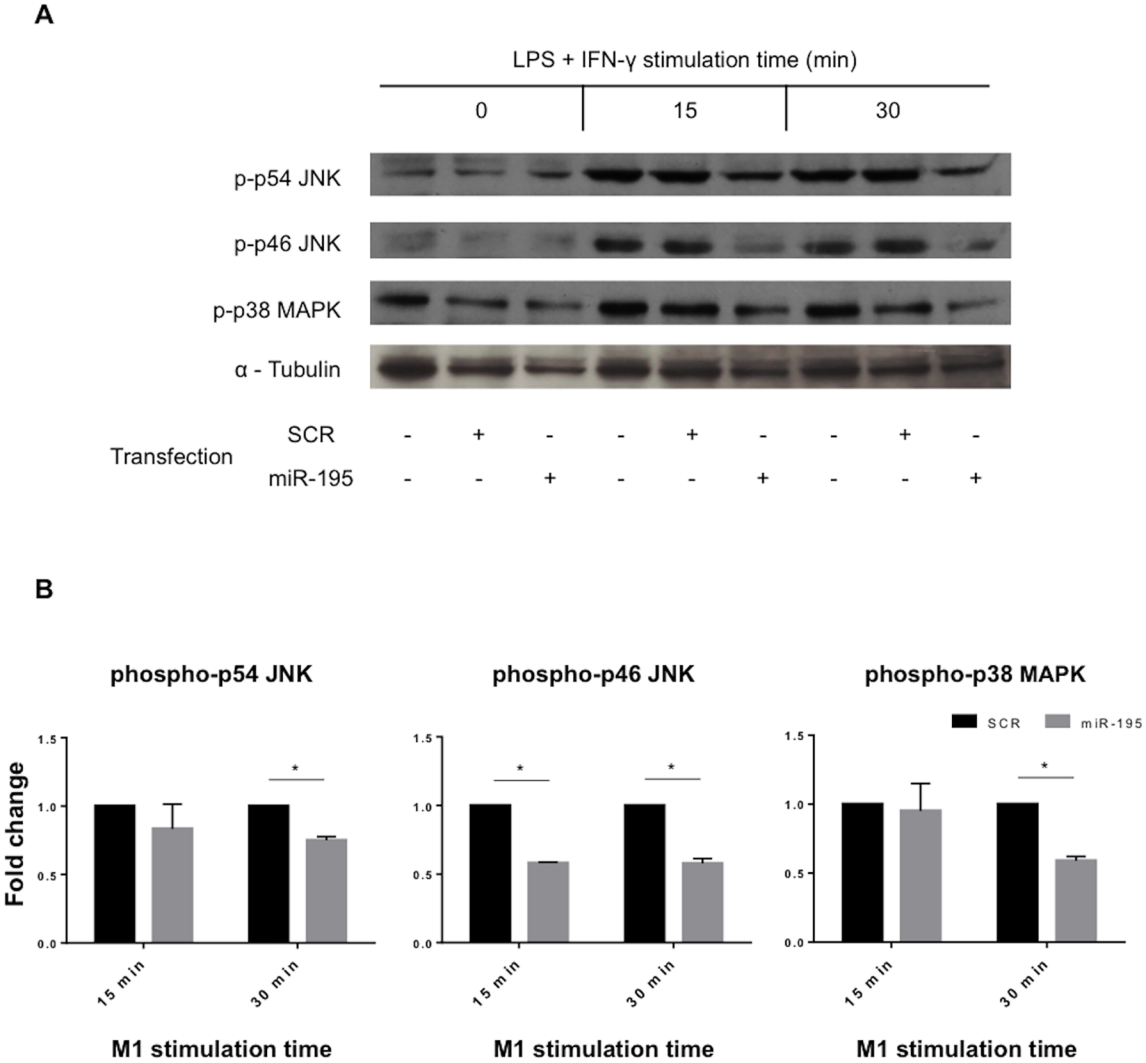
miR-195 decreases levels of p54 JNK, p46 JNK and p38 MAPK phosphorylated forms. A) Western blot shows protein levels of p54 JNK, p46 JNK and p38 MAPK phosphorylated forms in THP-1 macrophages after transfection with SCR control or miR-195 and M1 stimulation during 15 or 30 minutes. α-tubulin was used as normalizer. B) Quantification of phosphorylated p54 JNK, phosphorylated p46 JNK and phosphorylated p38 MAPK levels in two independent experiments using FIJI software. Statistical significance: *p<0.05.

Knowing that miR-195 influenced TLR2 expression, we further investigated the impact of miR-195 on TLR2 signaling pathway. Total and phosphorylated levels of p54 JNK, p46 JNK and p38 MAPK downstream kinases were analyzed upon pro-inflammatory stimulation. Differentiated THP-1 macrophages were stimulated with the M1 pro-inflammatory stimuli of LPS and IFN-γ at different time points. The peak phosphorylation levels of p54/p46 JNK were found to be around 15 and 30 min and of p38 MAPK around 5 and 15 minutes (S4A Fig). Next, levels of total and phosphorylated proteins were measured in non-transfected, SCR and miR-195 overexpressing cells. Results are illustrated in Fig 3A, and show that phosphorylation levels of p54 and p46 JNK and p38 MAPK are all significantly downregulated in miR-195-overexpressing cells, which indicates an active repression of TLR2 downstream effectors. Specifically, 30 min after M1 stimulation, phospho-p54 and phospho-p38 levels show a reduction of 25% (p<0.05) and 41% (p<0.05), respectively, in miR-195-overexpressing THP-1 macrophages (Fig 3B). Moreover, phospho-p46 was found to be significantly downregulated by 41% (p<0.05) in macrophages transfected with miR-195 compared with the respective SCR controls, after M1 stimulation for 15 and 30 minutes (Fig 3B). Nonetheless, differences in levels of total forms of p54 JNK, p46 JNK and p38 MAPK were not observed (S4B Fig), indicating that these proteins are not miR-195 direct targets. In conclusion, miR-195 impacts inflammatory pathways through downregulation of active phosphorylated forms of p54 JNK, p46 JNK and p38 MAPK proteins downstream of TLR2.

### Increased expression of miR-195 in macrophages decreases production of pro-inflammatory cytokines

**Fig 4:**
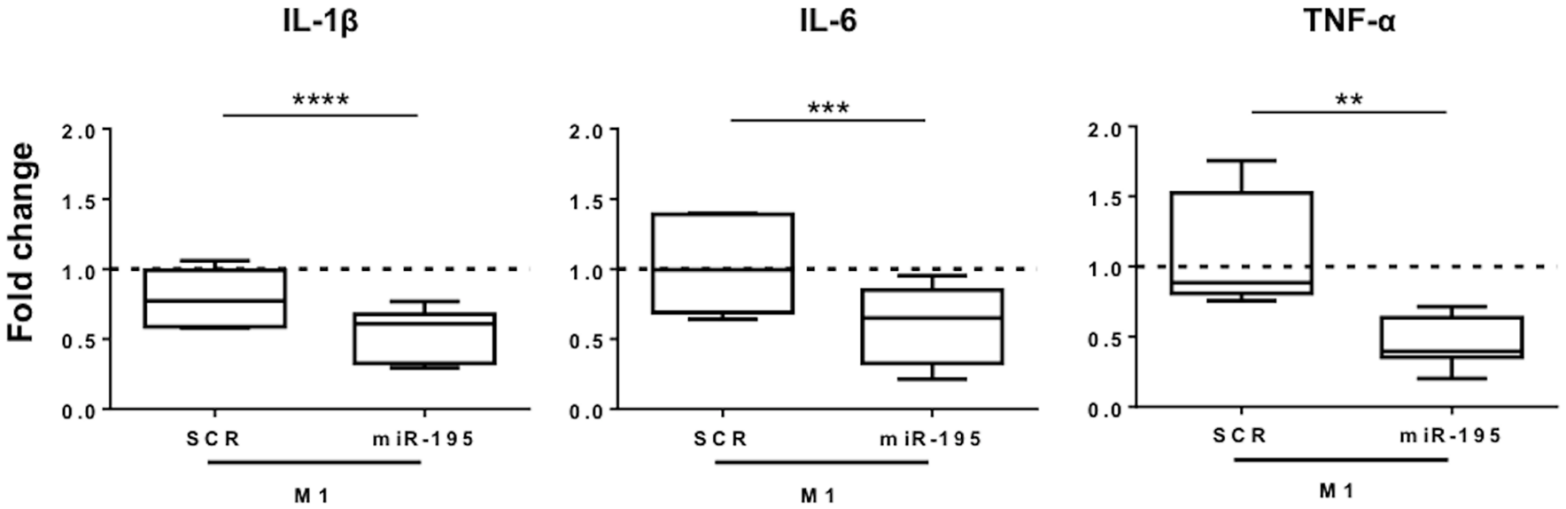
Levels of IL-1β, IL-6 and TNF-α secreted by macrophages after miR-195 overexpression upon pro-inflammatory stimulation. miR-195 mimic was transfected into THP-1 macrophages before stimulation with LPS and IFN-γ. After 24h, cell culture medium was collected and levels of IL-1β, IL-6, and TNF-α were determined by ELISA. Graph show results relative to non-transfected, M1-stimulated THP-1 macrophages. Three independent experiments were performed in triplicate. Statistical significance: **p<0.01, ***p<0.001, ****p<0.0001.

To assess the functional consequences of miR-195 interference with the TLR2 inflammatory pathway, we evaluated the cytokine secretion profile of macrophages. Supernatants of THP-1 macrophages transfected with miR-195 in pro-inflammatory conditions were collected 24 hours after stimulation, and analyzed by ELISA. Results showed that miR-195-macrophages produced significantly reduced amounts of IL-1β (p<0.0001), IL-6 (p<0.001) and TNF-α (p<0.01) pro-inflammatory cytokines compared with SCR control cells (Fig 4).

### miR-195 expression impacts inflammatory and atherosclerosis associated pathways

**Fig 5:**
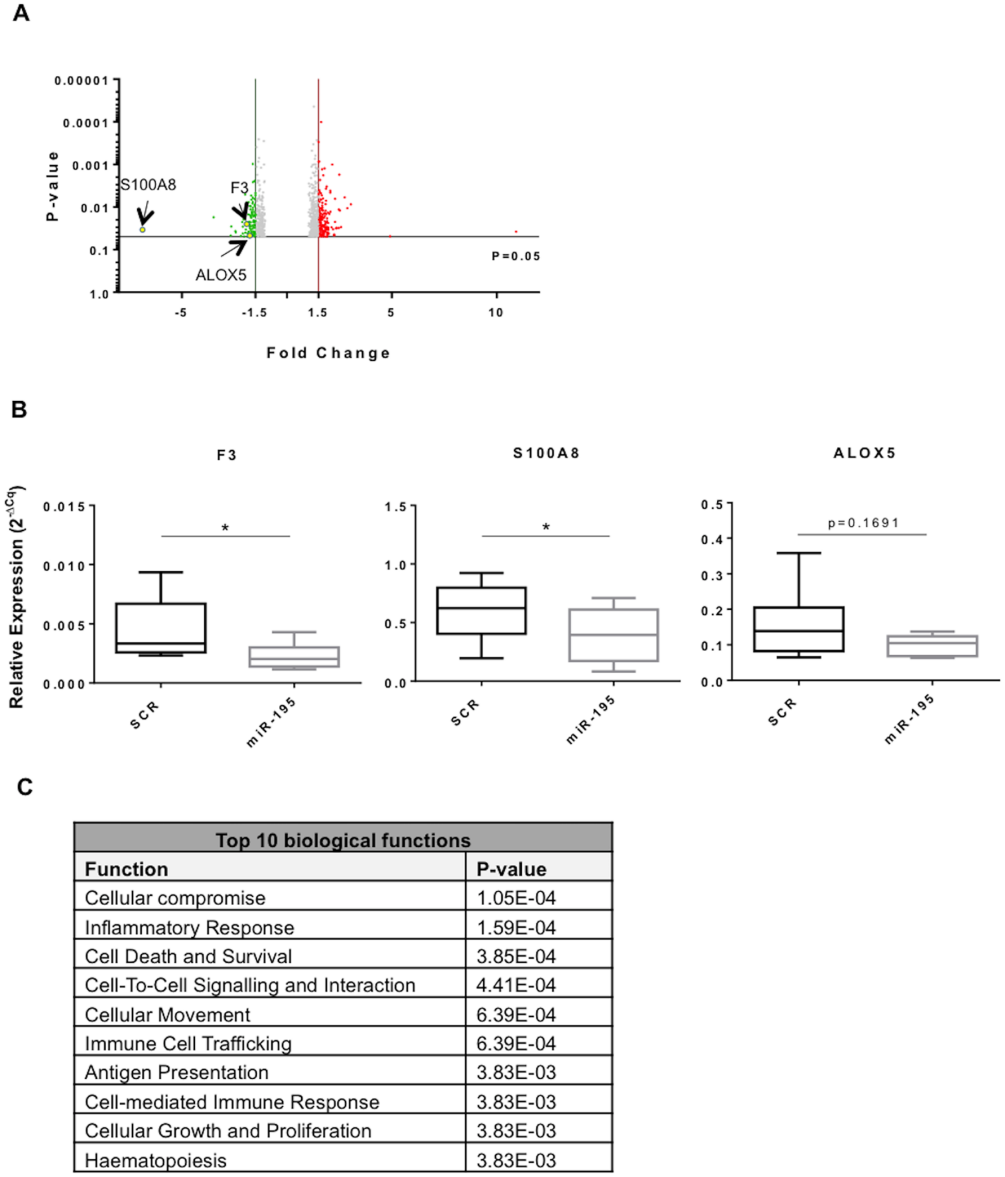
miR-195 downregulated genes are associated with inflammatory and cell recruitment mechanisms. A) Volcano plot of statistical significance against fold change representing most deregulated genes between miR-195 and SCR-transfected THP-1 macrophages. Green dots represent downregulated genes (fold change <-1.5); red dots represent upregulated genes (fold change > 1.5). F3, S100A8 and ALOX5 involved in atherosclerosis signaling appear highlighted. B) Measurement of F3, S100A8 and ALOX5 expression levels by RT-qPCR (n = 4). C) List of the 10 most prominent biological functions of genes downregulated by miR-195, determined by IPA. Statistical significance: *p<0.05.

To further identify relevant genes and pathways targeted by miR-195, the expression profile of THP-1 macrophages overexpressing miR-195 was compared with SCR-transfected cells. Bioinformatics analysis of the expression levels of the 92 genes significantly downregulated (fold change<-1.5, P-value<0.05) by miR-195 in THP-1 macrophages revealed that atherosclerosis signaling (P-value = 0.0147) is one of the top canonical pathways, as a result of F3, S100A8 and ALOX5 downregulation (fold change: -1.93, -6.87 and -1.65, respectively), three genes known to be involved in important mechanisms that contribute to disease pathophysiology (Fig 5A) [30,31]. Importantly, RT-qPCR results confirmed that F3 and S100A8 were significantly downregulated in macrophages overexpressing miR-195 compared with SCR-transfected macrophages (p<0.05). Regarding ALOX5, although not statistically significant, its expression levels tend to be downregulated by miR-195 as well (Fig 5B).

Moreover, important processes in atherosclerosis such as inflammatory response, cell-to-cell signaling and interaction, and immune cell trafficking are comprised among the most prominent biologic functions predicted to be affected by the most downregulated mRNAs in THP-1 macrophages overexpressing miR-195 (Fig 5C).

### Expression of miR-195 in macrophages inhibits smooth muscle cells recruitment and migration profile

In advanced stages of atherosclerosis, pro-inflammatory macrophages promote the recruitment of VSMC to the lesioned area, which also become lipid loaded foam cells, producing pro-inflammatory cytokines [32]. Thus, the impact of miR-195 overexpression in THP-1 macrophages, under pro-inflammatory stimuli, on HUASMC recruitment was evaluated using an *in vitro* recruitment model.

**Fig 6:**
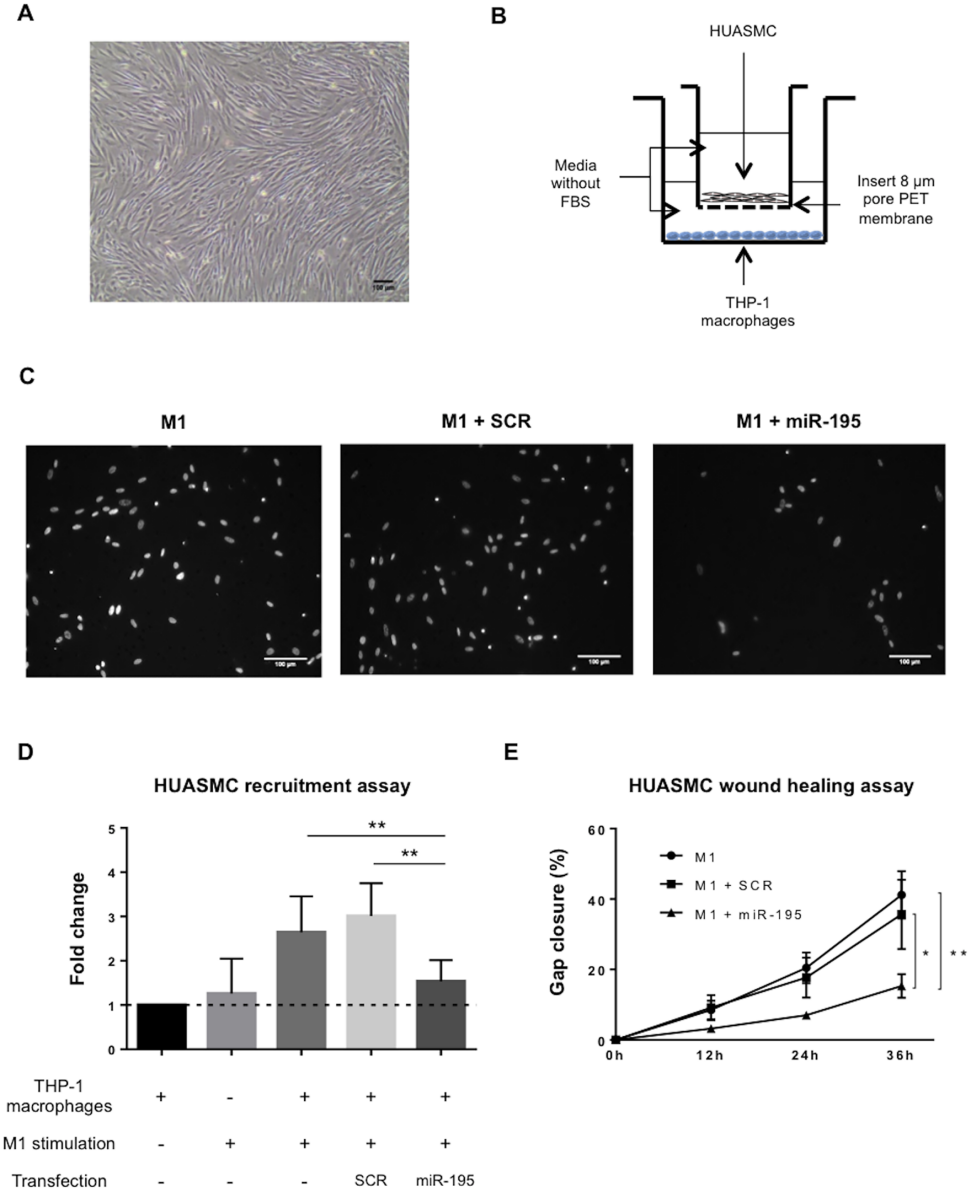
miR-195 reduces the capability of macrophages to recruit human umbilical artery smooth muscle cells and impair their migratory profile. A) HUASMC after 5 day culture in a collagen type I pre-coated flask (magnification of 50X). Scale bar 100 μm. B) Schematic representation of the transwell system set up for the migration assay. HUASMC were allowed to migrate for 24h over differently stimulated THP-1 macrophages. C) Representative inverted fluorescence microscope images of recruited HUASMC nuclei upon co-culture with macrophages subjected to different stimulation conditions. Scale bar 100 μm. D) HUASMC recruitment in different conditions. Column 1—non-stimulated THP-1 macrophages; Column 2 –No THP-1 macrophages, only M1 stimuli (LPS + IFN-γ); Column 3—M1 stimulated THP-1 macrophages; Column 4—M1 stimulated THP-1 macrophages transfected with SCR; and Column 5—M1 stimulated THP-1 macrophages transfected with miR-195. Two independent experiments were performed in triplicates (Mean ± SD). E) HUASMC migration profile over 36 hours in culture with conditioned media from differently stimulated THP-1 macrophages (Mean ± SEM, n = 4). Timepoint 0h was used as reference to calculate the percentage of gap closure. Statistical significance: *p<0.05, **p<0.01.

HUASMC were grown in a collagen coated surface to better mimic the *in vivo* growth environment of these cells (Fig 6A). We further characterized HUASMC cells by flow cytometry to determine levels of HUASMC surface markers [33]. CD90 was used as positive marker, while CD10 and CD34 (endothelial marker) were used as negative markers. Results clearly show high levels of CD90 (99.9% CD90^+^) and no expression of CD10 or CD34, in HUASMCs (S5A Fig). Furthermore, inverted fluorescence microscopy results show, as expected, positive expression of HUASMC markers, α-SMA and vimentin, two important SMC structural proteins (S5b Fig).

HUASMC were seeded in transwell inserts (top compartment) and co-cultured over differently stimulated THP-1 macrophages (bottom compartment) (Fig 6B). Results show that miR-195-THP-1 macrophages in pro-inflammatory conditions have significantly less capability to recruit HUASMC compared with non-transfected (p<0.01) and SCR-transfected pro-inflammatory THP-1 macrophages (p<0.01) (Fig 6C and 6D). Specifically, high expression levels of miR-195 in THP-1 macrophages reduce cell capability to recruit HUASMC by 40% (versus M1 non-transfected) and 50% (versus M1 SCR-macrophages) (Fig 6D). Importantly, miR-195 overexpression in macrophages under pro-inflammatory conditions is able to decrease HUASMC recruitment to similar levels of macrophages in a non-pro-inflammatory environment (Fig 6D).

To further confirm the impact of miR-195 in macrophages paracrine effect, we addressed HUASMC migration in an *in vitro* wound healing assay. Interestingly, HUASMC cultured in the presence of conditioned media from miR-195-M1-macrophages migrated significantly less than those cultured with conditioned media from M1 macrophages (p<0.01, 36 hours) or SCR-M1-macrophages (p<0.05, 36 hours) (Fig 6E).

## Discussion

miR-195 has been shown to control major cellular processes, including cell cycle progression, proliferation, migration and invasion, and cell differentiation [34,35]. In the cardiovascular system, miR-195 regulates VSMC proliferation, migration and cytokine secretion profile, and prevents neointimal formation [19]. Importantly, macrophages and VSMCs are major players in the pathogenesis of atherosclerotic vascular diseases. Activated pro-inflammatory macrophages promote pro-atherogenic functions of VSMCs [11]. For this reason, we hypothesized and evaluated if the anti-atherogenic miR-195 could act through macrophages by inhibiting their pro-inflammatory action and conditioning the communication with VSMCs.

Herein, we describe for the first time that miR-195 levels are increased in IL-10-treated human primary macrophages and further investigated miR-195 as a potential tool to inhibit mechanisms that promote M1 macrophage polarization. Macrophages can be polarized towards different phenotypes, depending on the microenvironment they encounter, and miRNA expression profiles have been explored to understand in which extent alterations in miRNA levels influence the immune functions of polarized macrophages [36]. For instance, miR-125a-5p inhibits the classical macrophage activation and decreases secretion of some inflammatory cytokines (IL-6 and TNF-α) [16,37], and miR-146a inhibits the activation of TLR4-dependent signaling pathways [16].

TLRs recognize distinct damage-associated molecular patterns and play a critical role in inflammation and immune cell regulation [38]. Activation of TLR-dependent signaling pathways promotes the consecutive phosphorylation of several protein kinases, leading to activation of MAP kinases (JNK, p38 MAPK and ERK) and activating the NF-κB transcription factor complex. Subsequently, transcription factors in the nucleus downstream the TLR signaling cascade are activated and induce the production of pro-inflammatory cytokines, such as IL-1β, IL-6, IL-8 and TNF-α [39,40]. In this study, we investigated how miR-195 impairs the activation of these pathways, analyzing TLR receptors and downstream molecules. TLR2 is known to be overexpressed in macrophages after LPS recognition and in pro-inflammatory conditions [41,42], acting as a key player in inflammation and atherosclerosis progression [24,43]. Furthermore, the regulation of its expression through miR-19 and miR-105 has been described by other authors [44,45]. Our results show, for the first time, that the expression of TLR2 is regulated by miR-195, as its levels are significantly reduced in miR-195-transfected THP-1 macrophages polarized towards M1 phenotype. Although a TLR2 mRNA::miRNA-195 direct interaction for the predicted binding site was not found, we do not exclude the hypothesis of an interaction in other region of the TLR2 mRNA. Moreover, the effect could be indirect through miR-195 binding to TLR2 regulators and transcription factors.

Although JNK isoforms (p54, p46) and p38 MAPK total protein levels were not altered, the activated/phosphorylated p54 JNK, p46 JNK and p38 MAPK forms were significantly decreased by miR-195. Remarkably, levels of IL-1β, IL-6 and TNF-α were reduced, which indicates that miR-195 efficiently inhibits the pro-inflammatory TLR2 signalling pathway, specifically p38 MAPK and JNK pathways, reinforcing the hypothesis that this specific miRNA acts as an anti-inflammatory regulator in macrophages. Moreover, expression of other genes associated with inflammatory pathways was found deregulated. Therefore, miR-195 can be used as a tool to counterbalance and reverse the effect of the induced M1 pro-inflammatory profile in macrophages.

In advanced states of atherosclerotic lesions, macrophages secrete high levels of pro-inflammatory cytokines and chemokines, which perpetuate the inflammatory process and promote migration of VSMC into the intima [46]. Sequentially, VSMC proliferate and secrete pro-fibrotic mediators, resulting in the fibrous cap formation. At some stages, this fibroproliferative response is associated with a protective physiologic response to injury, designed to wall off the injurious agent, and then to assist in resolution of the injury [47]. However, atherosclerotic VSMC are described to switch to a macrophage-like phenotype characterized by lipid over-ingestion, which leads to VSMC apoptosis and, consequently, to a necrotic core formation and weakening of the fibrous cap [32,47,48]. Thus, it is of crucial importance to limit both VSMC migration and the phenotypic switch.

To mimic *in vitro* the VSMC migration profile upon interaction with macrophages and the crosstalk between macrophages and VSMC in the atherogenic environment, we performed a transwell (using porous PET membranes) and a wound healing assay [49]. Interestingly, both assays showed that macrophages overexpressing miR-195 drastically lose their ability to promote VSMC recruitment in a pro-inflammatory environment. Although further experiments are needed to understand which mediators are changed by miR-195 and how do they affect VSMC-macrophages crosstalk, this is the first evidence that modulation of macrophage phenotype through miR-195 has a paracrine effect on VSMC.

In conclusion, miR-195 impacts macrophage polarization status, favoring an anti-inflammatory phenotype even in pro-inflammatory conditions, which leads to a reduction in the recruitment of VSMC. This suggests miR-195 as a potential tool to prevent and potentially treat atherosclerotic lesions and other cardiovascular diseases.

## Supporting information

S1 Fig Human primary monocyte culture, differentiation into macrophages, and polarization. A) Brightfield microscopy image of macrophages differentiated from isolated blood monocytes at day 10 of culture (magnification of 200X). Scale bar 50μm. B) Flow cytometry analysis of CD14 marker in macrophages (right panel). Cells labeled with matching isotype were used as negative control (left panel). C) Representative image of flow cytometry results for CD86 and CD163 markers in macrophages stimulated with LPS, IL-10 or control (non-stimulated cells).
(TIF)

S2 Fig Transfection efficiency of miR-195 mimics in THP-1 macrophages. Transfection efficiency was evaluated by RT-qPCR and compared with SCR control (n = 3). Statistical significance: ****p<0.0001.
(TIF)

S3 Fig Intracellular TLR4 levels in THP-1 macrophages. Intracellular TLR4 levels were measured by flow cytometry (n = 3). Statistical significance: *p<0.05.
(TIF)

S4 Fig Quantification of protein p54 JNK, p46 JNK and p38 MAPK levels. A) Representative western blot showing protein phosphorylated levels following LPS and IFN-γ stimulation at different time points (0, 5, 15, 30, 60, 120 minutes). B) Total protein expression of JNK isoforms and p38 MAPK was evaluated by western blot and levels were normalized to α-tubulin.
(TIF)

S5 Fig HUASMC characterization by flow cytometry and inverted fluorescence microscopy. A) Characterization of HUASMC by flow cytometry. Histograms represent flow cytometry results for CD10, CD34 (negative markers) and CD90 (positive marker). B) Evaluation of HUASMC markers by inverted fluorescence microscopy. Cells show positive staining for α-SMA (on top) and vimentin (on bottom) markers. Microscope images were obtained using a 40x oil objective. Scale bar 20 μm.
(TIF)

S1 Table Primers used for RT-qPCR. (DOCX)

S2 Table Sequence of mature human miR-195-5p according to miRBase, primers used to generate PGL3-constructs for luciferase assays and to generate deletions in the predicted miRNA-binding site. Restriction site for endonuclease is underlined.
(DOCX)
