# Supplementary figures and images for: miR-195 inhibits macrophages pro-inflammatory profile and impacts the crosstalk with smooth muscle cells
Source: PLoS One. 2017 Nov 22;12(11):e0188530. doi: 10.1371/journal.pone.0188530 (PMC5699821; doi:10.1371/journal.pone.0188530)

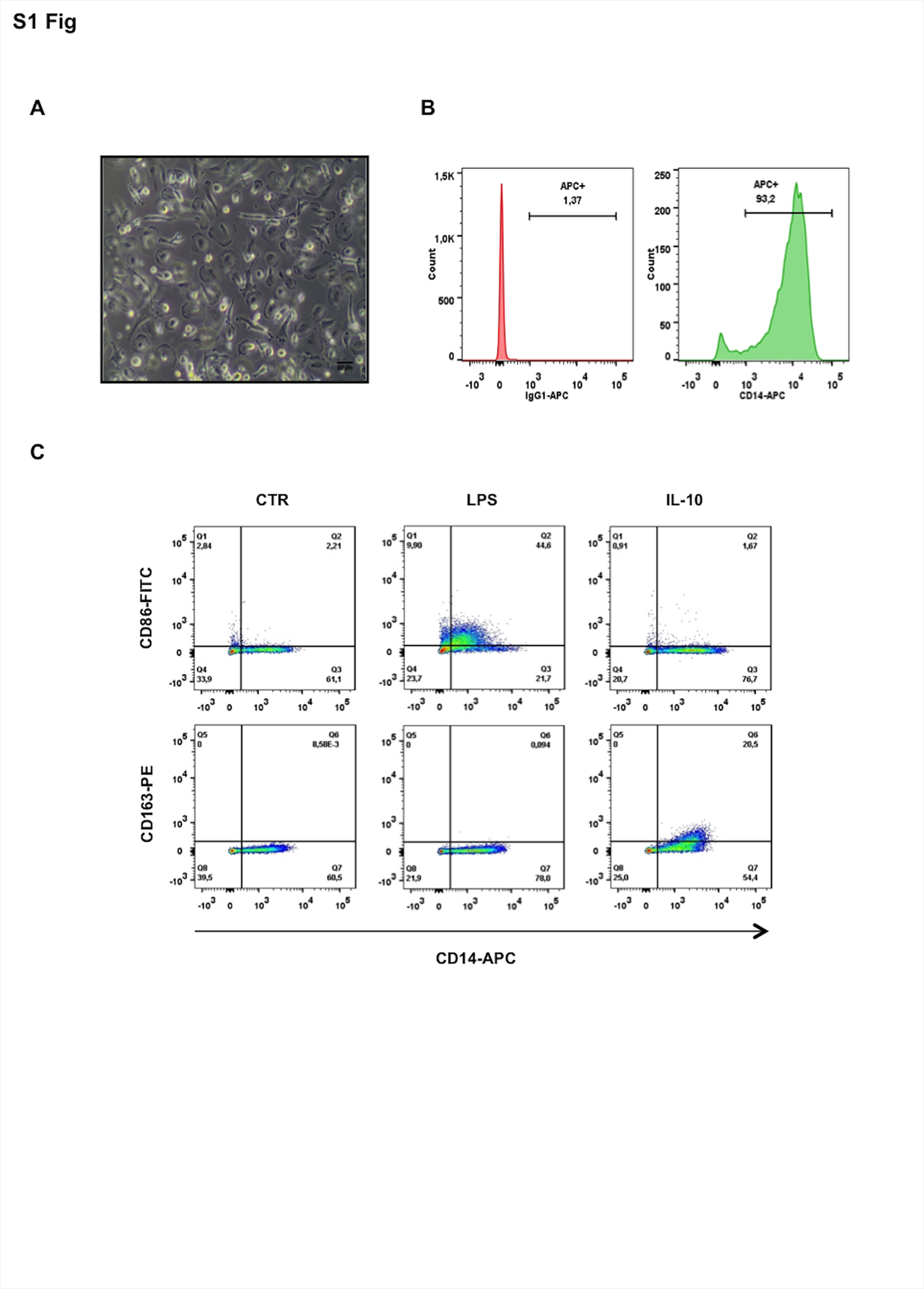

Supplement: S1 Fig — A) Brightfield microscopy image of macrophages differentiated from isolated blood monocytes at day 10 of culture (magnification of 200X). Scale bar 50μm. B) Flow cytometry analysis of CD14 marker in macrophages (right panel). Cells labeled with matching isotype were used as negative control (left panel). C) Representative image of flow cytometry results for CD86 and CD163 markers in macrophages stimulated with LPS, IL-10 or control (non-stimulated cells). (TIF) [file pone.0188530.s001.tif]

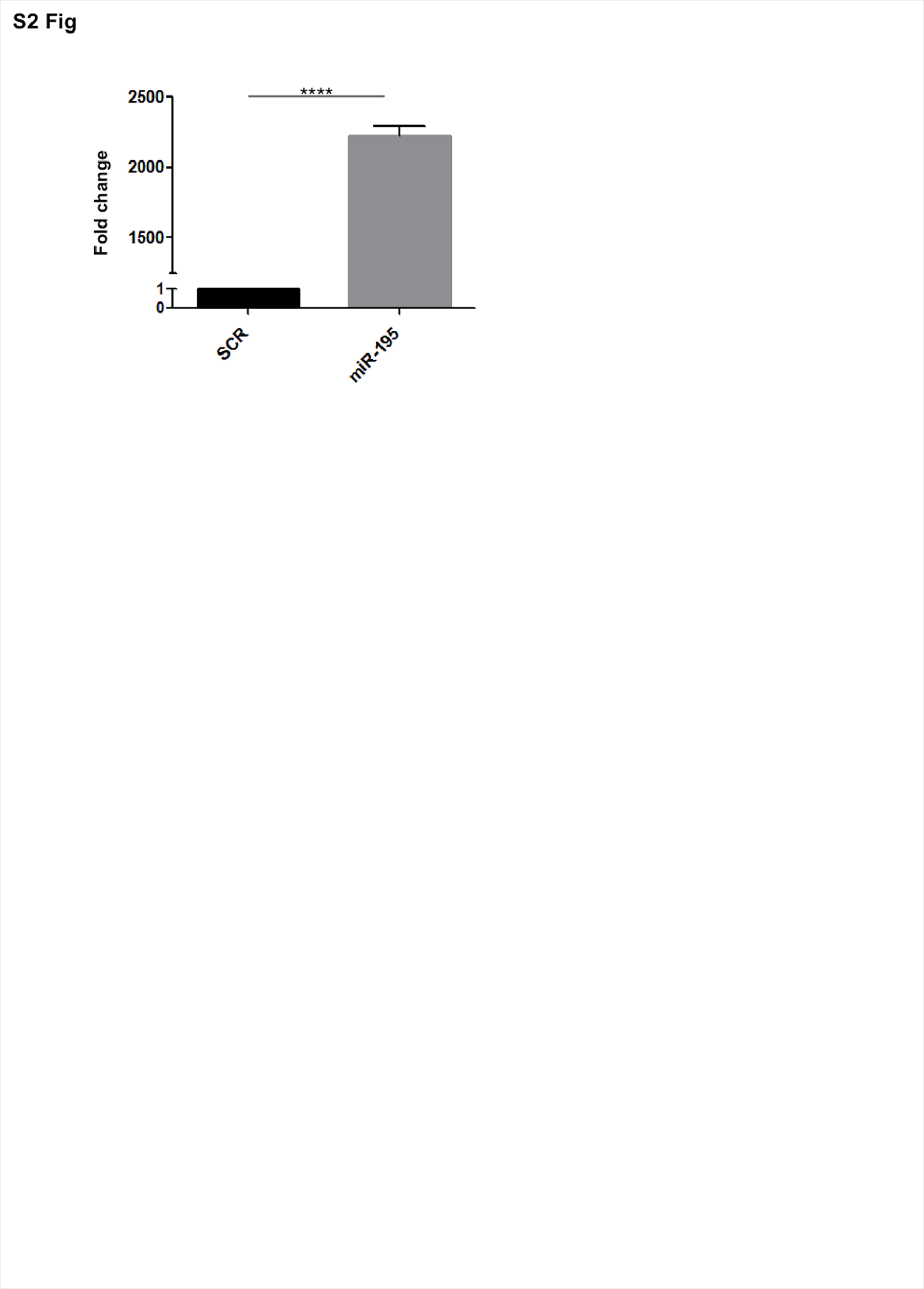

Supplement: S2 Fig — Transfection efficiency was evaluated by RT-qPCR and compared with SCR control (n = 3). Statistical significance: ****p<0.0001. (TIF) [file pone.0188530.s002.tif]

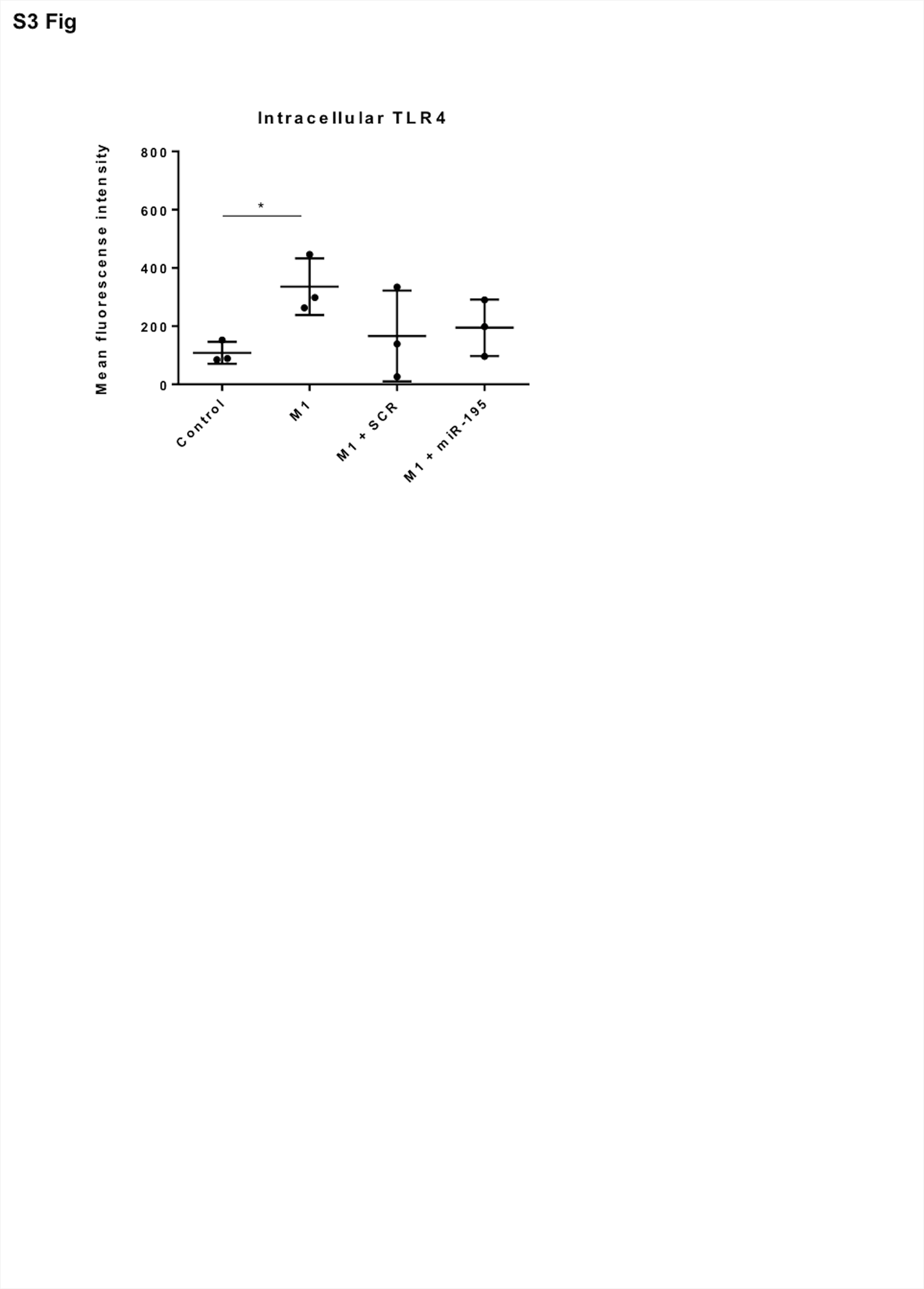

Supplement: S3 Fig — Intracellular TLR4 levels were measured by flow cytometry (n = 3). Statistical significance: *p<0.05. (TIF) [file pone.0188530.s003.tif]

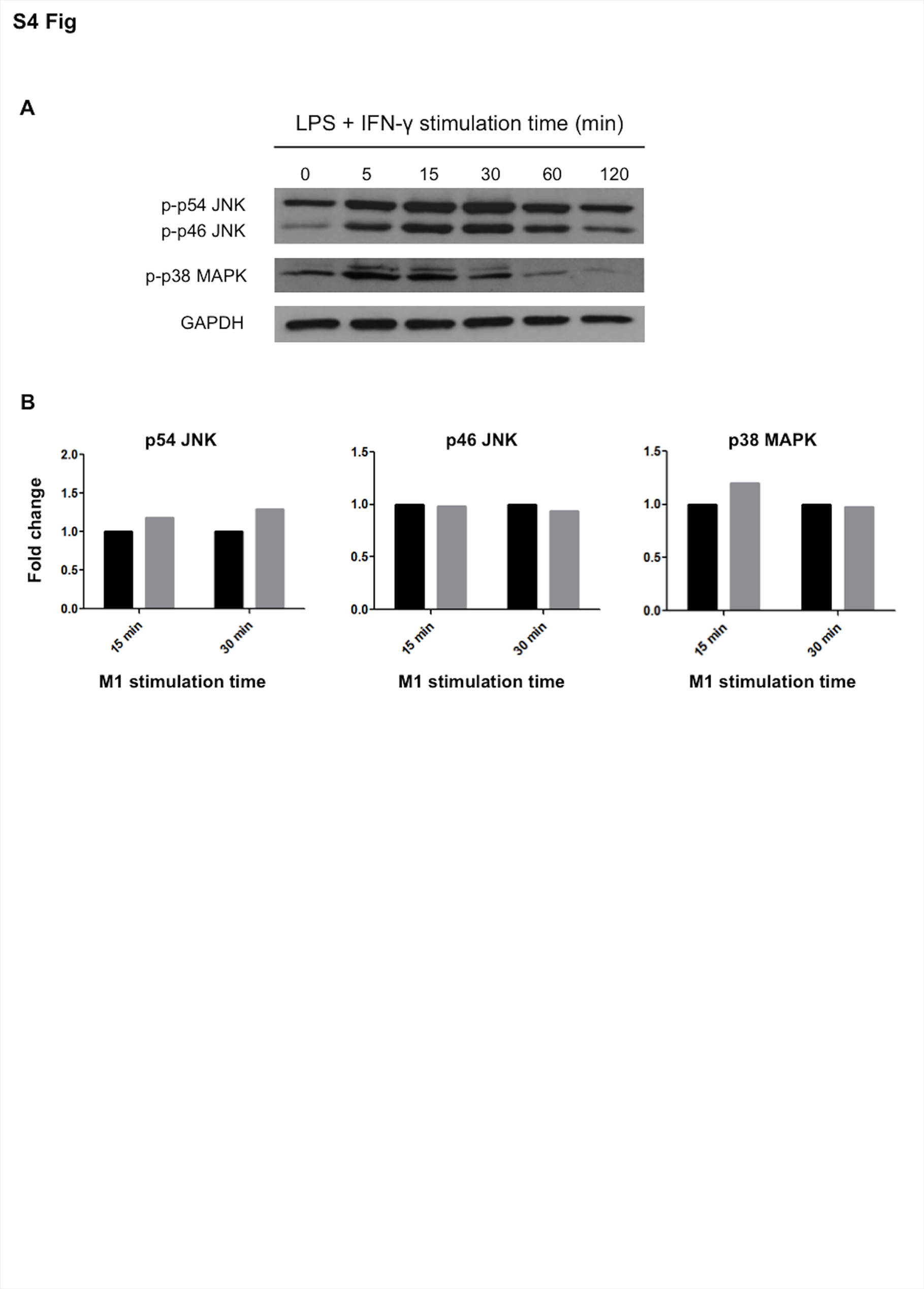

Supplement: S4 Fig — A) Representative western blot showing protein phosphorylated levels following LPS and IFN-γ stimulation at different time points (0, 5, 15, 30, 60, 120 minutes). B) Total protein expression of JNK isoforms and p38 MAPK was evaluated by western blot and levels were normalized to α-tubulin. (TIF) [file pone.0188530.s004.tif]

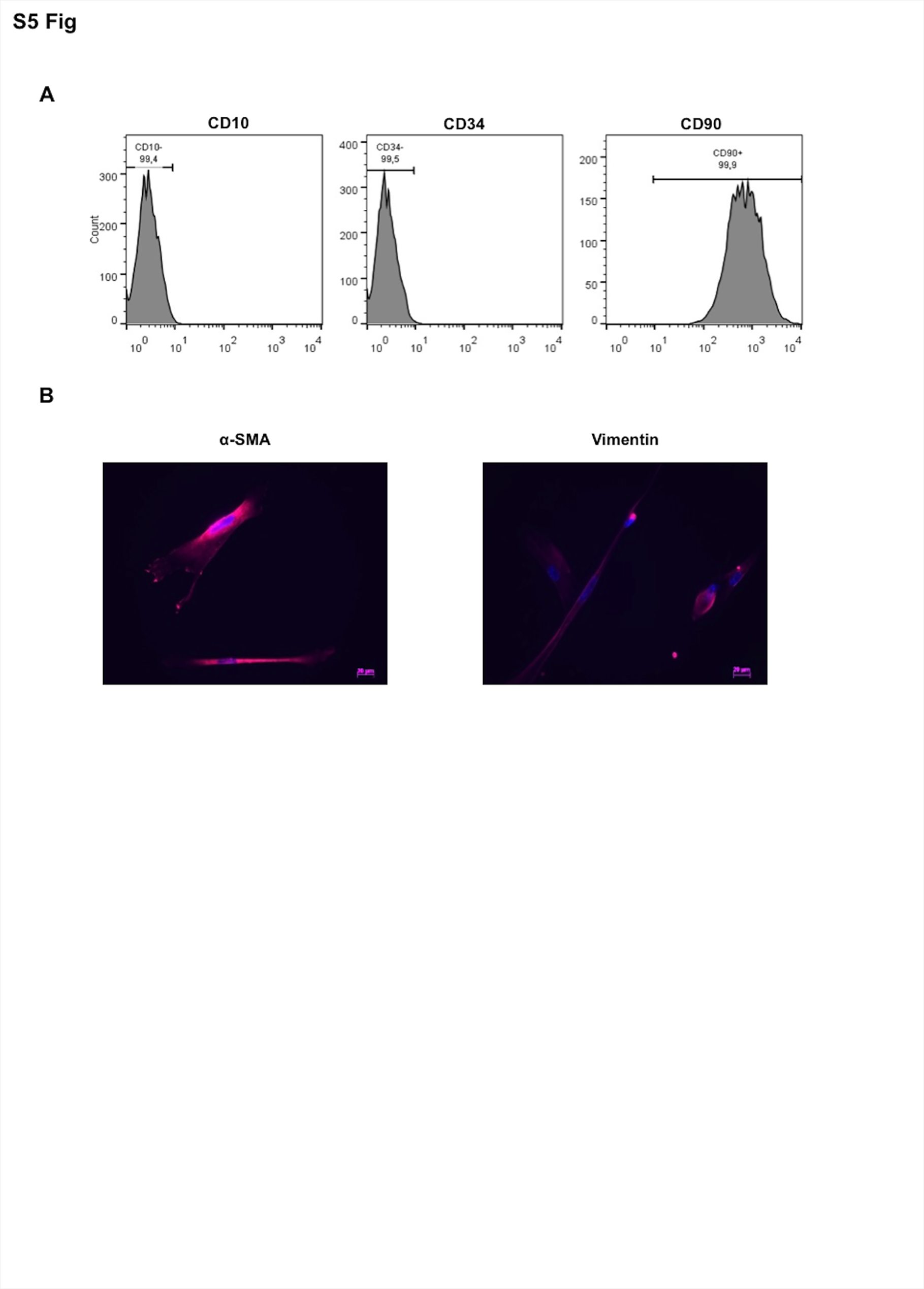

Supplement: S5 Fig — A) Characterization of HUASMC by flow cytometry. Histograms represent flow cytometry results for CD10, CD34 (negative markers) and CD90 (positive marker). B) Evaluation of HUASMC markers by inverted fluorescence microscopy. Cells show positive staining for α-SMA (on top) and vimentin (on bottom) markers. Microscope images were obtained using a 40x oil objective. Scale bar 20 μm. (TIF) [file pone.0188530.s005.tif]
