# Supplementary material for: miR-195 inhibits macrophages pro-inflammatory profile and impacts the crosstalk with smooth muscle cells
Source: PLoS One. 2017 Nov 22;12(11):e0188530. doi: 10.1371/journal.pone.0188530 (PMC5699821; doi:10.1371/journal.pone.0188530)
Supplement: S1 Table — (DOCX) [file pone.0188530.s006.docx]

**S1 Table – Primers used for RT-qPCR.**

| **Primer** | **Sequences** |
| --- | --- |
| S100A8_Fw | 5’-ATTTCCATGCCGTCTACAGG-3’ |
| S100A8_Rv | 5’-TGGCTTTCTTCATGGCTTTT-3’ |
| F3_Fw | 5’-GAATGTGACCGTAGAAGATG-3’ |
| F3_Rv | 5’-CACTGAAACAGTAGTTTTCTCC-3’ |
| ALOX5_Fw | 5’-CTCAAGCAACACCGACGTAAA-3’ |
| ALOX5_Rv | 5’-CCTTGTGGCATTTGGCATCG-3’ |
| GAPDH_Fw | 5’-CCTCAAGATCATCAGCAAT-3’ |
| GAPDH_Rv | 5’-CCATCCACAGTCTTCTGGGT-3’ |
