# Supplementary material for: miR-195 inhibits macrophages pro-inflammatory profile and impacts the crosstalk with smooth muscle cells
Source: PLoS One. 2017 Nov 22;12(11):e0188530. doi: 10.1371/journal.pone.0188530 (PMC5699821; doi:10.1371/journal.pone.0188530)
Supplement: S2 Table — Restriction site for endonuclease is underlined. (DOCX) [file pone.0188530.s007.docx]

**S2 Table - Sequence of mature human miR-195-5p according to miRBase, primers used to generate PGL3-constructs for luciferase assays and to generate deletions in the predicted miRNA-binding site.** Restriction site for endonuclease is underlined.

| **Mature microRNA** | **Sequences** |
| --- | --- |
| hsa-miR-195-5p | 5’-UAGCAGCACAGAAAUAUUGGC-3’ |
| **Primers for PGL3 construct** |  |
| pGL3_TLR2_XbaI_Fw | 5’-CGTCTAGATGTGACTCTCCATCCCATGT-3’ |
| pGL3_TLR2_XbaI_Rv | 5’-CGTCTAGACAGGCCCACATCATTTTCAT-3’ |
| **Primers for mutagenesis** |  |
| TLR2_Mut_Fw | 5’-CCCGTGAGCAGGATGAACAGAGCACAGC-3’ |
| TLR2_Mut_Rv | 5’-GCTGTGCTCTGTTCATCCTGCTCACGGG-3’ |
